# State-dependent reward circuit modulation gates social buffering of stress

**DOI:** 10.21203/rs.3.rs-9441690/v1

**Published:** 2026-05-21

**Authors:** Urszula Skupio, Djemila Compaore, Jaehan Kwon, Nekhama Riznyk, Alexander Z Harris

**Affiliations:** 1 Department of Psychiatry, Columbia University, College of Physicians and Surgeons, New York, NY, USA; Area Neuroscience, New York State Psychiatric Institute, New York, NY, USA.

## Abstract

Social experiences can profoundly shape emotional regulation and promote resilience to stress. Although the presence of companions is well-known to buffer the impact of stress in humans and rodents^1–5^, whether and how social interactions restore the disruptions in reward processing commonly produced by stress remains unknown. Here, we developed a reward-based behavioral paradigm to study social buffering. Brief interaction with a familiar, unstressed partner following acute restraint stress restored reward-seeking behavior in both female and male C57BL6/J mice. Our findings indicate that buffering is an active process driven by the stressed individual, rather than a passive outcome of social interaction. Ventral tegmental area (VTA) GABAergic, but not dopaminergic, neurons play a key role in mediating this effect. Critically, temporally precise inhibition of VTA GABA neuron activity is sufficient to convert a non-buffering interaction with a novel partner into a buffering experience, whereas identical inhibition delivered outside of a social context is ineffective. These results reveal a previously unrecognized role for socially elicited VTA GABA neuron activity in gating the response to stress and position the stressed individual’s behavior as a key determinant of resilience. Given the central role of stress-induced reward deficits in psychiatric disorders, these findings have broad implications for understanding resilience and the development of novel therapies.

## Main:

Stress-related mental health disorders have risen dramatically in recent years, affecting millions worldwide^6–8^. Impaired reward processing^9^ is a core symptom that cuts across multiple diagnoses, including major depressive disorder^10,11^ and schizophrenia^12,13^ and is often treatment resistant^14,15^. Stress can directly cause reward-seeking deficits and disrupt key components of the mesolimbic reward circuit, including the ventral tegmental area (VTA)^16^.

Vulnerability to stress-induced psychiatric symptoms is further exacerbated by social isolation^17^. This was strikingly evident during the COVID-19 pandemic, when widespread isolation coincided with a global increase in affective disorders^18,19^. In contrast, social support can mitigate the impact of stress on mental health^2,20^. Social buffering, where the presence of conspecifics mitigates stress responses, is a robust form of social support which is conserved across multiple species^1–5,21,22^. However, since the direct physical interactions between distressed individuals and their partners have rarely been studied^5, but see 23–25^, the specific behavioral and neural components that determine successful buffering remain poorly defined.

Prior work has implicated stress- and social-responsive neuromodulators and circuits in the communication of affective states and social buffering in rodents, including corticosteroids, oxytocin, and regions such as the paraventricular hypothalamus, amygdala, and prefrontal cortex^23,26–37^. However, despite its role in social interactions^38^ and stress-mediated disruptions of reward process^16^, very little is known about whether social buffering acts directly on the brain’s reward circuitry^39^. Specifically, it remains unknown whether and how social interactions modulate neuronal activity within the VTA circuit to preserve reward responsiveness under stress.

Here, we developed a novel, reward-based behavioral paradigm to investigate social buffering in mice while recording and manipulating the activity of VTA dopamine (DA) and GABA neurons, two reward circuitry populations that play key roles in reward processing and stress^16^. We show that brief interactions with a familiar, unstressed partner following acute restraint stress restores reward seeking in both sexes. Remarkably, affiliative behaviors initiated by the stressed mice, rather than the partner, were the strongest predictors of social buffering. This effect is mediated by context specific VTA GABA, but not dopamine neuron activity, revealing an unexpected role for GABAergic signaling within the mesolimbic circuit in promoting stress resilience through social experience.

### Familiar social interaction buffers stress-induced deficits in reward seeking.

**Extended Data Fig. 1.**
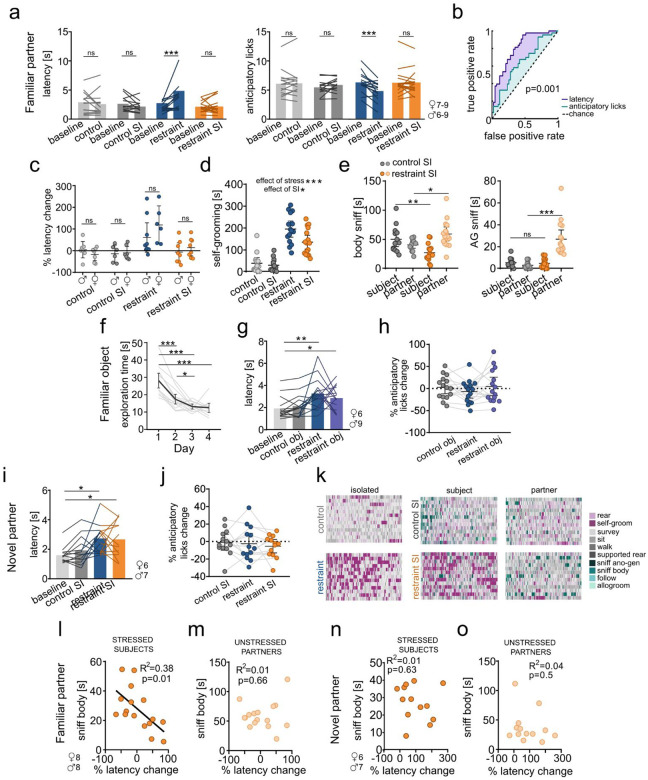
Behavioral features of stress buffering and non -buffering interactions. **(a)** Latency to respond to reward-predicting tones (left) and anticipatory licks (right) were altered following restraint stress and restored by brief social interaction (SI) with a familiar partner. **(b)** Receiver operating characteristic (ROC) curves for latency and anticipatory licking in control versus restraint conditions, indicating greater discriminative performance for latency. **(c)** No sex differences were observed under control or post-stress conditions. **(d)** Self-grooming increased following restraint stress and was reduced by SI. **(e)** In subject mice, body sniffing decreased following stress, whereas it increased in partner mice (left). Anogenital sniffing by partner mice toward stressed individuals was also increased (right). **(f)** Habituation to a familiar object was reflected by decreased exploration time across days. **(g)** Interaction with a familiar object did not alter the stress-induced increase in latency to respond to reward predicting tones. **(h)** Anticipatory licking was not significantly affected by stress or interaction with a familiar object. **(i)** Interaction with a novel partner did not alter the stress-induced increase in latency. **(j)** Anticipatory licking was not significantly affected by stress or interaction with a novel partner. **(k)** Behavioral patterns during interaction with a novel partner under control and post -stress conditions. Each row represents behavioral annotations from an individual mouse over a 10 min session. **(l,m)** Body sniffing by stressed subjects toward familiar partners **(l)**, but not by partners toward stressed subjects **(m)**, predicts subsequent reward-seeking behavior. **(n,o)** During interaction with a novel partner, body sniffing by neither subjects **(n)** nor partners **(o)** predicts subsequent reward-seeking behavior **(a–d,k–m)** Data correspond to SI with a familiar partner; **(e–g)** to familiar object interaction; **(h–j,n–o)** to SI with a novel partner. Significant differences detected in post hoc analysis are marked with *p<0.05, **p<0.01, ***p<0.001; ns, not significant. Data presented as individual data points with mean ± 95%CI. Statistical tests are provided in Supplementary Table 2.

**Fig. 1.**
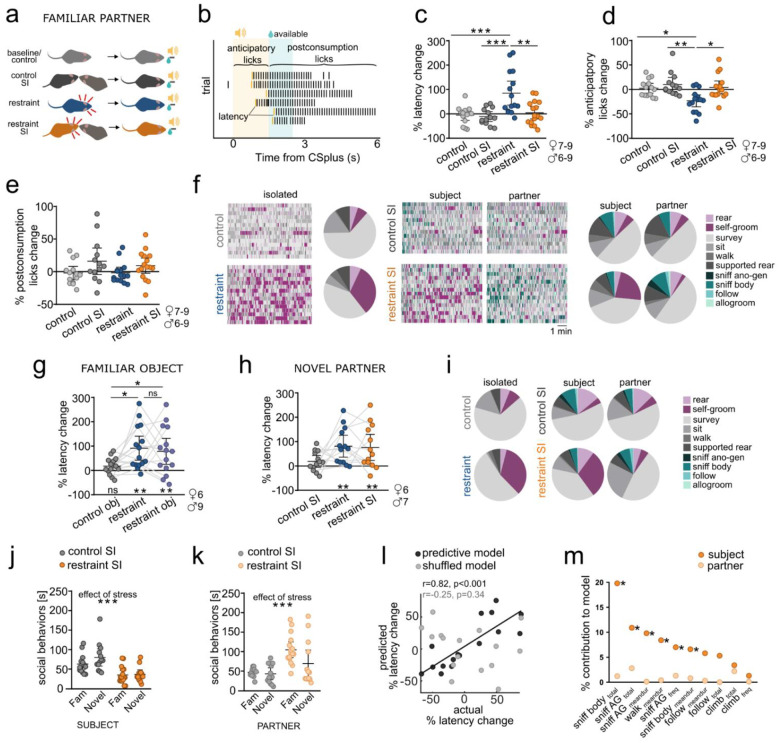
Familiar social interaction buffers stress-induced deficits in reward seeking. **(a)** Experimental design. Unstressed or restraint-stressed mice were either kept isolated or engaged in brief social interaction (SI) with a familiar partner, followed by a cued reward task. **(b)** Example raster plot of several trials from the cued reward task. Each trial begins with a 1.5 s tone. Latency is defined as the time to the first lick from tone onset. Licks during the tone, when reward is not available, are defined as anticipatory licks, and licks during and after the 1 s reward availability period are defined as post-consumption licks. **(c)** Latency to respond to reward-predicting tones was increased following restraint stress, an effect reversed by brief SI with a familiar partner. **(d)** Anticipatory licking was reduced following restraint stress and was restored by brief SI with a familiar partner. **(e)** Post-consumption licking was unaffected by stress or SI. **(f)** Distinct behavioral patterns emerged following restraint and during SI. **(g)** Interaction with a familiar object did not alter the stress-induced increase in latency. **(h)** Interaction with a novel partner did not buffer the stress-induced increase in latency. **(i)** Behavioral patterns during interaction with a novel partner. **(j)** Subject-initiated, as well as **(k)** partner-initiated social behaviors were similar in duration toward familiar and novel interaction partners across control and stress conditions. **(l)** A predictive model based on behavioral features during post-stress familiar partner interaction correlated with observed latency changes, whereas a shuffled control model did not. **(m)** A random forest model identified subject-initiated prosocial behaviors as the most predictive features, with minimal contribution from partner behaviors. Significant differences detected in post hoc analysis are marked with *p<0.05, **p<0.01, ***p<0.001; ns, not significant. Data presented as mean ± 95%CI. Statistical tests are described in Supplementary Table 1.

To investigate whether social interaction ameliorates stress-induced deficits in reward seeking, we developed a behavioral paradigm combining acute stress, social exposure, and reward seeking behavior. Mice were first trained on a cued-reward task, and upon reaching performance criteria, underwent 2–4 daily testing sessions consisting of a 30-minute exposure to either a familiar environment or acute restraint stress, followed by a 10-minute recovery period in either social isolation or with a familiar partner, and finally a cued-reward test (Fig 1a). During the test, we measured latency to respond to reward-predictive cues, anticipatory licking during the cue when reward was not yet available, and consummatory licking after reward delivery (Fig. 1b). As we previously reported^40^, acute restraint stress increased response latency and reduced anticipatory licking (Fig. 1c–d). Remarkably, 10 minutes of post-stress interaction with a familiar partner fully restored these behaviors to baseline levels (Fig. 1c–d, Extended Data Fig. 1a). We did not observe effects of either stress or social interaction on consummatory licking rates (Fig. 1e). To quantitatively assess which behavioral metric most robustly captured stress effects across these conditions, we performed receiver operating characteristic (ROC) analysis across baseline and stress sessions in the familiar partner, familiar object, and novel partner experiments. This analysis demonstrated that reward retrieval latency more robustly discriminated stress from control states than anticipatory licking (Extended Data Fig. 1b). As we observed no significant differences between males and females in any condition (Extended Data Fig 1c), data from both sexes were pooled for all further experiments.

We observed striking differences in behavioral patterns during 10-minute recovery period across conditions (Fig 1f). Consistent with previous reports^41^, the most notable effect of acute stress was a marked increase in self-grooming, which was observed in both isolation and social context (Fig 1f, Extended Data Fig 1d). Following 30 minutes in a familiar environment, the behavioral profiles of subject and partner mice were largely similar but diverged following acute stress exposure. Notably, unstressed partners presented increased prosocial interactions towards stressed subjects, particularly body sniffing and anogenital sniffing (Fig 1f, Extended Data Fig 1e) indicating a directed social response to stress exposure in the interaction partner^23,26,42^.

We subsequently set out to determine the specificity of the buffering effect. To isolate the contribution of familiarity, we replaced the familiar partner with a familiar inanimate object (Extended Data Fig 1f). In contrast to the familiar social partner, the presence of the familiar object failed to mitigate stress-induced deficits in reward seeking: stressed mice exhibited prolonged response latencies in both conditions (Fig. 1g, Extended Data Fig 1g–h). We next tested whether any social interaction, even with an unfamiliar partner, could provide buffering. Interaction with an unfamiliar sex-matched partner failed to attenuate the effects of stress, with latencies elevated following restraint, irrespective of social presence (Fig. 1h, Extended Data Fig 1i–j). One possible explanation for the lack of buffering might be reduced social interaction between unfamiliar animals. However, behavioral scoring during the 10 min post-stress period revealed that overall patterns of social behavior (Fig 1i, Extended Data Fig 1k), including total time spent in social contact (Fig 1j–k), were comparable between familiar and novel partner conditions.

Since the partner mice spend more affiliative time with the stressed vs non-stressed mice, we hypothesized that this pro-social behavior of the familiar partner facilitates social buffering. To address this question, we trained a supervised machine learning model using behavioral features of both subject and partner mice extracted from the 10 min post-stress interaction period (Fig 1f). Model performance was evaluated using a leave-one-mouse-out cross-validation scheme. The neural network model trained on this dataset successfully predicted latency changes, with a significant correlation between predicted and actual latency change (Fig 1l). By contrast, decoupling behavioral features from reward seeking behavior by shuffling the data resulted in predictions that did not correlate with actual changes in latency (Fig 1l). To identify the most informative behavioral predictors, we applied a complementary model-based feature importance analysis using the same feature set and performed 1,000 permutation tests against shuffled data. Surprisingly, this analysis revealed that all six features that significantly outperformed chance reflected behaviors initiated by the stressed subject, rather than by the unstressed partner (Fig. 1m). Notably, five of these six features represent social behaviors of stressed subjects, suggesting that social buffering might be a process predominantly driven by active affiliative engagement from the stressed individual. Among these, body sniffing emerged as the strongest predictor (Fig. 1m). This finding was further supported by linear regression analyses across stressed subjects and unstressed partners in both familiar and novel pairings: body sniffing duration predicted reward-seeking behavior exclusively in stressed subjects interacting with familiar partners (Extended Data Fig. 1l–o). Together, these results identify affiliative behavior of the stressed individual, particularly body sniffing, as a key predictor of social buffering and demonstrate that its effectiveness depends on partner familiarity.

### VTA GABA, but not dopamine, neurons selectively respond to social buffering determinants.

**Extended Data Fig. 2.**
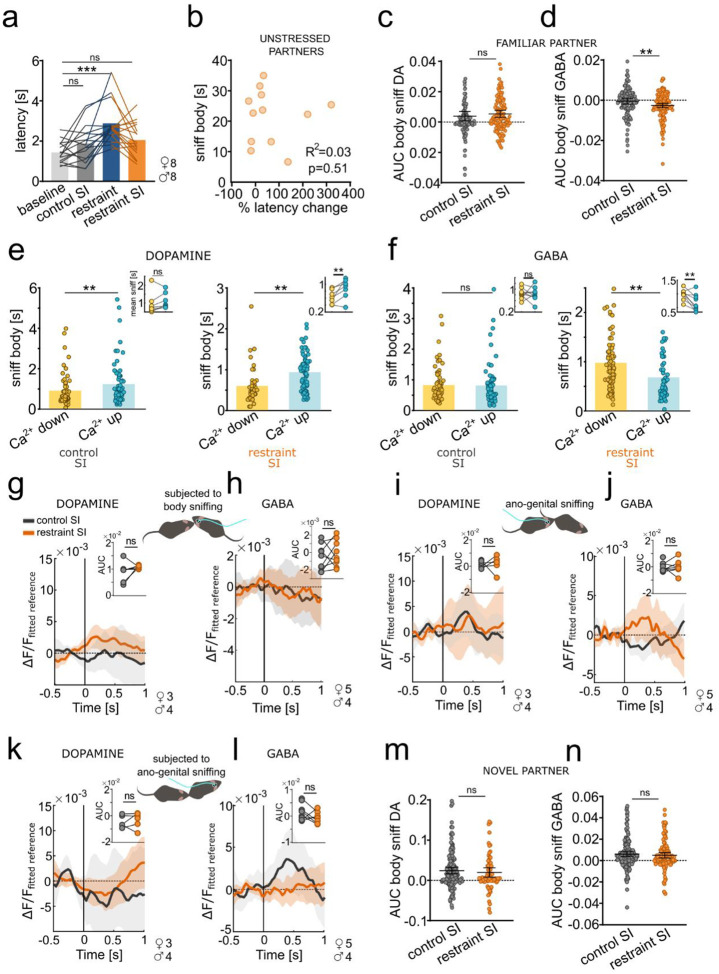
Event specific calcium dynamics in VTA DA and GABA neurons during social interaction. **(a)** Latency to respond to reward-predicting tones increased following restraint stress and was restored by brief SI with a familiar partner. **(b)** Body sniffing by unstressed partners toward stressed subjects did not predict subsequent reward-seeking behavior **(c,d)** During interaction with a familiar partner, area under the curve (AUC) during body-sniffing events was similar between control and stress conditions in VTA dopamine neurons **(c)**, but was reduced following stress in VTA GABA neurons **(d)**. Each dot represents one event. **(e)** In VTA dopamine neurons, body-sniffing bouts were longer during events with higher calcium signals compared to lower signals, under both control (left) and restraint (right) conditions. **(f)** In VTA GABA neurons, body-sniffing duration did not differ between events above or below baseline under control conditions (left), but following restraint, longer bouts were associated with decreased calcium signals (right). **(g,h)** No changes in calcium activity were observed in VTA DA **(g)** or GABA **(h)** neurons when mice were subjected to body sniffing by their partners. **(i-l)** No changes in calcium activity were observed in VTA DA or GABA neurons when stressed mice initiated (DA, **i**, GABA, **j**) or were subjected to (DA, **k**, GABA, **l**) anogenital sniffing. **(g-l)** Calcium signals aligned to the onset of annotated behavior (time 0) and normalized to pre-event baseline (−0.5 to 0 s). Insets (top right) in **(e-l)** show mean responses per mouse. **(m, n)** During interaction with a novel partner, AUC during body sniffing events was similar between control and stress conditions in both DA **(m)** and GABA **(n)** neurons. **(a-l)** correspond to SI with a familiar partner **(m-n)** correspond to interaction with novel partner. Significant differences detected in post hoc analysis are marked with *p<0.05, **p < 0.01, ***p < 0.001; ns, not significant. Data presented as individual data points or mean ± 95%CI. Statistical tests are provided in Supplementary Table 2.

**Fig. 2.**
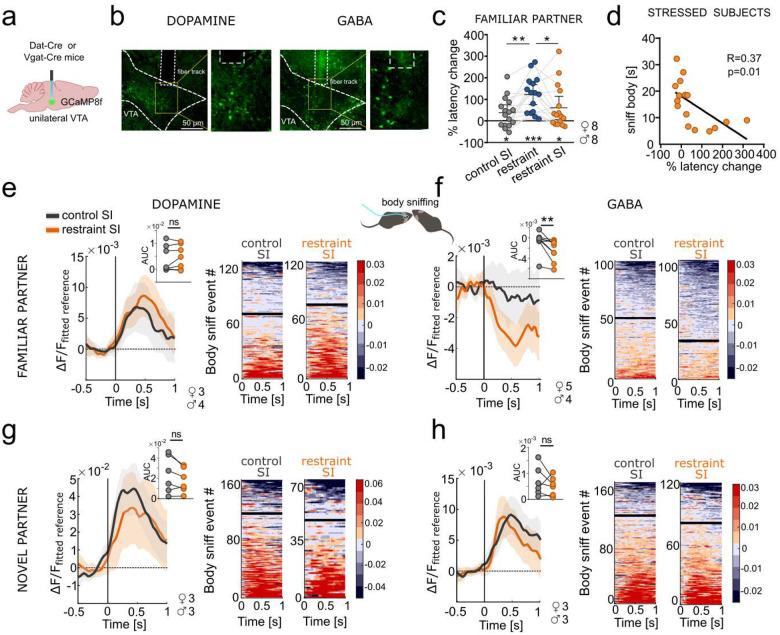
Distinct social response profiles of VTA dopamine and GABA neurons. **(a)** Schematic of the fiber photometry approach. **(b)** Example images showing GCaMP8f expression in the VTA of DA (left) or GABA neurons (right). **(c)** In fiber photometry mice, latency to respond to reward-predicting tones was increased following restraint stress and was restored by brief SI with a familiar partner. **(d)** Duration of body sniffing initiated by stressed subjects is predictive of their subsequent performance in cued-reward task. **(e)** Body sniffing behavior towards a familiar partner induced increased calcium signals of VTA DA neurons under both control and post-stress conditions. **(f)** Body sniffing did not alter VTA GABA calcium signals under control condition but resulted in significant decrease of calcium signal following restraint stress. **(g,h)** During interaction with an unfamiliar partner, calcium signals increased in both VTA dopamine **(g)** and GABA **(h)** neurons under control and post-stress conditions. **(e-h)** Calcium signals aligned to the onset of annotated body-sniffing behavior (time 0) and normalized to pre-event baseline (−0.5 to 0 s). Insets (top right) show mean responses per mouse. Heatmaps show all recorded calcium events. Black lines separate events with increased versus decreased calcium signals relative to the pre-event baseline. Significant differences detected in post hoc analysis are marked with *p<0.05, **p<0.01, ***p<0.001; ns, not significant. Data presented as mean ± 95%CI. Statistical tests are described in Supplementary Table 1.

We next set out to define the neural substrates underlying social buffering of stress. VTA DA neurons mediate reward processing^43^, social behaviors^44–46^, and are disrupted by stress^16^. In addition, recent evidence has highlighted the role that VTA GABA neurons play in these processes not only by locally modulating VTA DA^47^, but also by exerting DA independent effects^40,48^. We therefore tested the role of these neurons in social buffering. We recorded calcium activity in these two distinct populations^49^ by expressing Cre-dependent GCaMP8f in the VTA of *Dat*-Cre and *Vgat*-Cre mice (Fig. 2a–b). All fiber photometry recordings were obtained exclusively from the subject mice. These mice recapitulated the behavioral effects observed in non-implanted animals (see Fig. 1c), exhibiting reduced reward-seeking latency in the presence of a familiar partner (Fig. 2c, Extended Data Fig. 2a). Consistent with Fig. 1m (and Extended Data Fig. 1l–m), linear regression identified body sniffing initiated by stressed subjects, but not unstressed partners, as a predictor of reward-seeking latency (Fig. 2d, Extended Data Fig. 2b).

To examine the underlying neural dynamics, we aligned calcium signals to the onset of body sniffing. In VTA DA neurons, body sniffing of a familiar partner induced increases in calcium activity after both control and restraint conditions (Fig. 2e; Extended Data Fig. 2c, **Supplementary Video 1**). In contrast, VTA GABAergic neurons exhibited a state-dependent pattern: no modulation was observed during body sniffing after familiar environment exposure, whereas body sniffing after restraint stress was associated with a significant decrease in calcium signals (Fig. 2f; Extended Data Fig. 2d, **Supplementary Video 2**).

Given that longer subject-initiated body sniffing predicts social buffering (Fig. 1m, Extended Data Fig. 1l, Fig. 2d), we asked whether calcium dynamics within individual events were reflected in the duration of body-sniffing bouts. Calcium events were classified based on whether the mean signal during the event was above or below baseline. In VTA DA neurons, body-sniffing bouts were longer during events with increasing calcium signals compared to those with decreasing signals, after both control and restraint (Extended Data Fig. 2e). In contrast, after control exposure, increasing or decreasing VTA GABA calcium events did not differ in body-sniffing duration, while following restraint, body-sniffing bouts were longer during events with decreased GABA calcium signals (Extended Data Fig. 2f).

When mice were subjected to body sniffing by their partners, no significant calcium changes were detected in either DA or GABA neurons (Extended Data Fig. 2g,h). Similarly, alignment to anogenital sniffing events, whether subject-initiated or partner-initiated, did not reveal significant modulation in either population (Extended Data Fig. 2i–l). These findings indicate that neural responses were differentially engaged during active body sniffing initiated by the stressed subject.

Finally, we examined neural responses during interaction with a novel partner. As with familiar partner, social interaction with a novel mouse elicited robust calcium increases in DA neurons, independent of stress condition (Fig. 2g; Extended Data Fig. 2m). Strikingly, VTA GABA neurons displayed a markedly different calcium activity pattern depending on partner familiarity. During interaction with a familiar partner, GABA activity was either unmodulated (control) or suppressed (post-restraint, Fig. 2f). In contrast, interaction with a novel partner induced calcium increases in GABA neurons, and this effect was independent of stress (Fig. 2h; Extended Data Fig. 2n).

Collectively, these data indicate that VTA DA neurons are broadly engaged by social interaction regardless of familiarity or stress. By contrast, VTA GABA neurons activity is attuned to both the context (stress vs unstressed) and identity (familiar vs novel) of the social interaction. Notably, longer body-sniffing bouts were observed during events with decreased GABA calcium signals following restraint, linking this activity pattern to the key behavioral predictor of social buffering (Extended Data Fig. 2f). This dissociation in calcium dynamics during social interaction points to a potential role of GABAergic, rather than dopaminergic, neural activity in mediating social buffering.

### Causal role of VTA GABA neurons in mediating social buffering.

**Extended Data Fig. 3.**
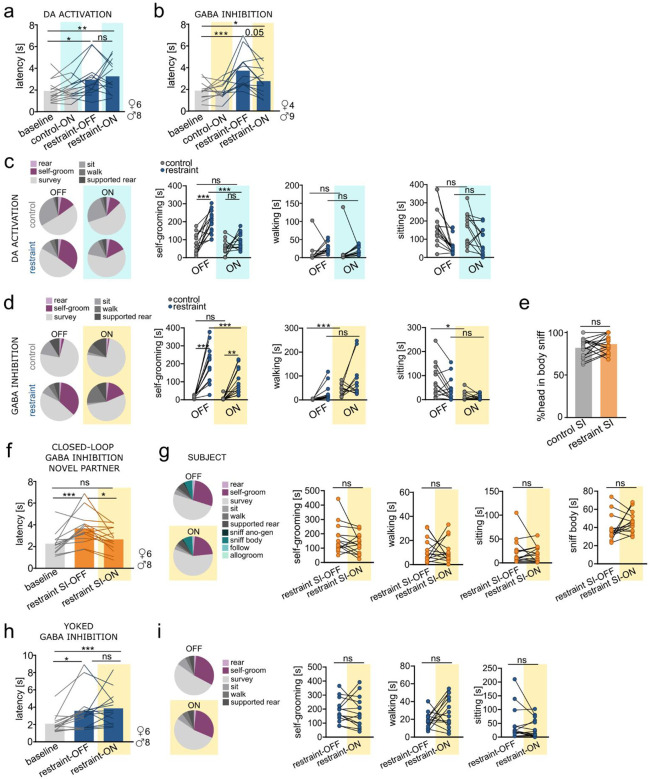
Behavioral characterization of optogenetic manipulations of VTA DA and GABA neurons. **(a)** Latency to respond to reward-predicting tones increased after restraint stress both without (OFF) and with VTA DA activation (ON). **(b)** Latency increased following restraint stress both without (OFF) and with VTA GABA inhibition (ON), with a trend toward reduced latency in the laser ON condition (P=0.05). **(c)** Behavioral annotations during 10 min sessions under control and post-stress conditions (left) show that VTA dopamine activation reduced post-stress self-grooming, without affecting walking or sitting (right). **(d)** Behavioral annotations (left) show that VTA GABA inhibition reduced post-stress self-grooming and increased walking while decreasing sitting under control conditions (right). **(e)** Head-region exploration during body-sniffing events, quantified in the fiber photometry cohort, indicates that the majority of events occur in the head region and are similar across control and stress conditions. **(f)** Latency to respond to reward-predicting tones increased following restraint during interaction with a novel partner, but was reduced when interaction was paired with closed-loop VTA GABA inhibition. **(g)** Behavioral annotations during 10 min interaction with a novel partner under post-stress conditions with or without closed-loop VTA GABA inhibition (left) revealed no differences in self-grooming, walking, sitting, or time spent body sniffing (right). **(h)** Latency increased following restraint and was not affected by yoked VTA GABA inhibition. **(i)** Behavioral annotations during 10 min post-stress sessions with or without yoked VTA GABA inhibition (left) revealed no differences in self-grooming, walking, or sitting (right). Significant differences detected in post hoc analysis are marked with *p<0.05, **p<0.01, ***p<0.001; ns, not significant. Data are presented as individual data points or as proportions (pie charts). Statistical tests are provided in Supplementary Table 2.

**Fig. 3.**
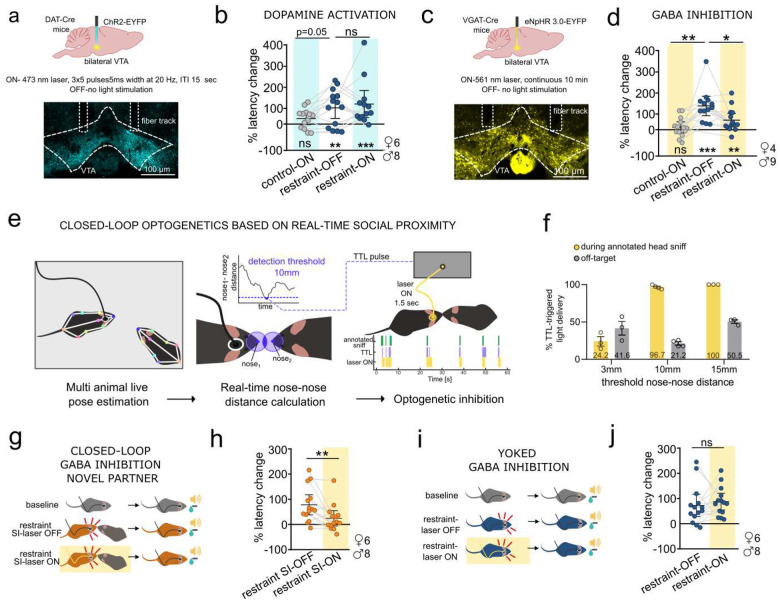
VTA GABA neurons mediate social buffering. **(a)** Schematic of VTA DA neuron activation (top), and example image showing ChR2 expression in the VTA DA neurons (bottom). **(b)** Optogenetic activation of VTA DA neurons did not alter stress-induced increase in latency to respond to reward-predicting tones. **(c)** Schematic of VTA GABA inhibition (top) and representative image showing eNpHR3.0 expression in VTA GABA neurons (bottom). **(d)** Continuous optogenetic inhibition of VTA GABA neurons during post-stress period reduced the stress-induced increase in latency to respond to reward-predicting tones. **(e)** Schematic of closed loop optogenetic approach. Freely moving mice were tracked in real time, and nose-nose distance was computed continuously. When a predefined threshold was crossed, a TTL pulse triggered a 1.5 s laser stimulation. **(f)** Comparison of different detection thresholds for triggering light stimulation during annotated head-sniffing events versus off-target events. **(g)** Closed loop experimental design. Mice were tested in pseudorandom order across baseline, post-stress interaction with a novel partner without light, and post-stress interaction with a different novel partner paired with closed-loop stimulation. (**h**) VTA GABA inhibition during episodes of social proximity with a novel partner reduced latency to respond to reward-predicting tones compared to interaction with a novel partner without light stimulation. **(i)** Yoked experimental design. Mice were tested in pseudorandom order across baseline, post -stress without light, and post-stress with yoked light stimulation. (**h**) Yoked VTA GABA inhibition did not alter stress-induced increase in latency to respond to reward-predicting tones. Significant differences detected in post hoc analysis are marked with *p<0.05, **p<0.01, ***p<0.001; ns, not significant. Data presented as mean ± 95%CI. Statistical tests are described in Supplementary Table 1.

To directly test the functional contribution of VTA DA and GABA neurons to social buffering, we mimicked buffering-associated activity patterns by activating DA neurons and inhibiting GABA neurons in the absence of a social partner (Fig. 2e–f). Optogenetically activating VTA dopamine neurons (Fig. 3a) during the 10-minutes interval after restraint failed to rescue stress-induced impairments in reward seeking, as reward retrieval latency remained indistinguishable from restraint sessions without light delivery (Fig. 3b; Extended Data Fig. 3a). In contrast, inhibiting VTA GABA neurons (Fig. 3c) during the same post-stress period significantly reduced reward-seeking latency, phenocopying the behavioral effects observed during social buffering (Fig. 3d; Extended Data Fig. 3b).

Interestingly, both VTA DA activation and VTA GABA inhibition reduced post-stress self-grooming behavior (Extended Data Fig. 3c–d). However, only inhibition of VTA GABA neurons restored reward-seeking performance, indicating that reductions in self-grooming do not account for the behavioral rescue. This dissociation is consistent with the empirical computational ethologic analysis, in which self-grooming was not among the top behavioral predictors of social buffering (Fig. 1m). In line with previous reports showing that suppression of VTA GABA activity increases locomotion^50^, our data showed that photoinhibition of VTA GABA neurons increased walking and reduced sitting under control condition (Extended Data Fig. 3d). Thus, although this sustained photo-inhibition across the 10-minute post-restraint period was sufficient to restore reward-seeking behavior, it likely constitutes a broad and non-physiological perturbation of circuit activity. While informative in globally establishing a causal role for VTA GABA neurons, it does not directly address whether modulations of social interaction-elicited activity in these neurons underlies social buffering.

To more precisely study this possibility, we implemented a closed-loop optogenetic strategy based on real-time social proximity. Using multi-animal live pose estimation, we computed the nose-nose distance between two freely moving mice and triggered 1.5 s light delivery whenever a predefined detection threshold was crossed (Fig. 3e, **Supplementary Video 3**). We focused on nose-nose proximity because quantitative analysis of body-sniffing events in the fiber photometry cohort (Fig. 2) revealed that approximately 85% of these events occurred in the head region rather than at the trunk (Extended Data Fig. 3e), making this metric a reliable proxy for affiliative investigation. To optimize detection specificity, we tested three distance thresholds (3 mm, 10 mm, and 15 mm) and quantified the proportion of light deliveries occurring during experimenter-annotated head sniffing versus off-target periods. A 10 mm threshold was selected for further tests, as it provided the best balance between sensitivity and specificity, with 96.7% of light deliveries occurring during annotated sniffing events and 21.2% occurring off target (Fig. 3f). This approach allowed for real-time, temporally precise inhibition of VTA GABA neurons triggered specifically by social proximity between freely moving mice.

As noted above, social interaction with a novel partner after stress does not provide stress buffering (Fig. 1i–j) and is associated with an increased rather than decreased VTA GABA neuron activity (Fig. 2h). We reasoned that if this difference in VTA GABA neuron activity patterns is causally responsible, closed-loop inhibition of VTA GABA neuron activity should transform a novel mouse interaction into a stress buffering experience. Stressed mice interacted with a novel partner either without light delivery or with closed-loop photoinhibition of VTA GABA neurons temporally restricted to ongoing social interaction (Fig. 3g). Strikingly, pairing brief VTA GABA inhibition with novel social interaction was sufficient to induce a buffering-like effect, significantly reducing reward-seeking latency compared to novel social interaction without light delivery (Fig. 3h, Extended Data Fig. 3f). Notably, this closed-loop manipulation did not produce the locomotor changes observed during continuous 10-minute VTA GABA inhibition, and body-sniffing behavior was comparable between light-off and light-on conditions (Extended Data Fig. 3g).

These results demonstrate that mimicking physiologic patterns of VTA GABA neuron inhibition during social interactions phenocopies the impact of social buffering on reward seeking. However, it remained unclear whether this was purely a consequence of inhibiting VTA GABA neurons (albeit for a shorter period) or represented a qualitative shift in the social interaction with the stranger mouse. If the former is true, then briefly inhibiting VTA GABA neurons should mitigate stress-induced blunted reward seeking even in the absence of a social partner. To answer this question, we conducted a yoked illumination experiment. In this paradigm, light delivery patterns were matched to those of a representative closed-loop animal but were delivered in the absence of a social partner (Fig. 3i). This yoked VTA GABA inhibition also did not produce the broader behavioral changes observed during continuous VTA GABA inhibition (Extended Data Fig. 3i), indicating that this manipulation avoided the widespread behavioral effects associated with sustained inhibition. Importantly, without the presence of a novel social partner, VTA GABA inhibition did not reproduce the stress-buffering effect (Fig. 3j, Extended Data Fig. 3h). These results indicate that VTA GABA inhibition must be temporally coupled to ongoing social interaction to alter reward-seeking behavior.

Collectively, these experiments establish a causal role for VTA GABA neurons in regulating stress-induced reward deficits, demonstrating that their inhibition is sufficient to restore behavior. Remarkably, brief VTA GABA inhibition delivered specifically during interaction with a novel partner was sufficient to convert a typically non-buffering interaction with a stranger into a stress buffering experience.

## Discussion

Social support is a key determinant of resilience, yet the mechanisms by which it confers its protective benefits remain unclear. Here, we show that social buffering represents an active state-dependent process driven by the stressed individual that dynamically modulates midbrain circuit activity. Specifically, we find that stressed mouse exploration of a familiar partner selectively inhibits VTA GABA neuron activity and reverses the impact of stress on reward seeking. These findings challenge the prevailing view that unstressed partners play the lead role in alleviating the deleterious effects of stress^5,24,25,51,52^ and provide a framework in which stress and social experience regulate reward seeking by gating circuit dynamics with temporal and behavioral specificity.

While it has long been known that familiar partners buffer stress more effectively than strangers^52–54^, the neural mechanism underlying this distinction has proved elusive. Here we show that similar social behaviors elicit distinct circuit responses depending on the state of the individual and the identity of the partner. Affiliative investigation of a familiar partner following stress selectively suppresses VTA GABA activity, yet the same behavior under control conditions, or during interactions with a novel partner, does not. These findings suggest that social behaviors do not have fixed neural or functional significance but instead are shaped by the organism’s internal state^55,56^. In contrast, social interaction uniformly engages VTA DA neurons, regardless of stress or partner familiarity and stimulating them did not restore reward-seeking behavior. Together, these results argue that the stress-alleviating effects of social interactions do not stem from the DA-dependent impact of simple rewards^57^, but instead require state-dependent regulation of inhibitory tone within the mesolimbic system.

Strikingly, by inhibiting VTA GABA activity specifically during social proximity we conferred buffering properties to an otherwise non-comforting interaction with a stranger. This effect was not recapitulated with identical patterns of inhibition delivered in the absence of social cues, indicating that circuit modulation alone is insufficient and must coincide with ongoing social cues to induce buffering effect. In line with this idea, similar observations have been made in other behavioral circuits. For example, in mice, optogenetic activation of the ventromedial hypothalamus elicits aggression towards other males, females and even an inanimate object, but fails to produce aggressive behavior in the absence of an attackable target^58^. Social interactions involve the integration of many complex types of cues, including olfactory, pheromonal, visual, somatosensory, and auditory. The specific combination of these signals were shown to shape social perception, influence decision-making, and ultimately determine behavioral outcomes during social encounters^59^. Interestingly, our data shows that closed-loop VTA GABA inhibition did not increase the overall time spent on social investigation, raising the possibility that this manipulation may modulate how ongoing social cues are processed rather than simply promoting more social engagement. Which of these cues are most relevant for social buffering, and how exactly they impinge on VTA GABA neurons, remains an interesting question for further investigations.

Together, our results establish behavioral and circuit mechanisms by which social interactions promote resilience in stressed individuals, positioning VTA GABA neurons as a critical interface between social experience, stress, and reward seeking. More broadly, they demonstrate that social buffering arises from the dynamic integration of internal state and ongoing social cues, revealing a general principle by which social interaction regulates reward circuit function under stress, and providing a framework for future interventions.

## Methods

### Animals

C57BL6/J (Jackson Laboratory, USA), DAT-Cre and VGAT-Cre male and female mice were used for different experiments of this project. Both sexes were used for all described experiments. All animals were bred and housed in the animal facility of the New York State Psychiatric Institute with controlled temperature of 21±2 °C, 30–60% humidity, in a 12h light/dark cycle (lights on at 7 am) with water and food ad libitum before the beginning of the experiments. Mice were 8–13 weeks of age at the time of surgical procedures and 10–22 weeks of age during behavioral testing. All familiar partners were same-sex siblings, housed together since weaning. All novel partners were age and sex matched to subject mice. All procedures were approved by New York State Psychiatric Institute Institutional Animal Care and Use Committee at Columbia University (authorization # NYSPI-1671-T1), in accordance with NIH guidelines.

### Cued-reward task training

To motivate reward-seeking behavior, mice were given ad libitum access to 2% citric acid (20 g/L, dissolved in water) for two nights prior to the start of training. Body weight was recorded prior to citric acid exposure and monitored daily throughout the training period. Mice were minimally handled during training. Behavioral training was conducted in a custom -built, rectangular, gray wooden chamber controlled by an Arduino Mega 2560 microcontroller and illuminated with red light (30–40 lux). Auditory cues (either a 1 kHz tone or 5–10 kHz white noise) were delivered via a buzzer (Digi-Key, MN, USA) and served as conditioned stimuli (CS). Reward delivery (5 μL of 0.1% saccharin) was controlled by a solenoid valve (The Lee Company, CT, USA), and licking behavior was detected using an infrared photo interrupter (Sparkfun, CO, USA). Each training session lasted 30 min and consisted of ~70 trials, with variable inter-trial intervals (17–33 s; mean ITI: 25 s). Mice were trained over 4 phases to associate auditory cues with reward availability. **Habituation** (1 day): Mice were allowed to lick freely at the spout, and reward was delivered contingent on each lick. A 3 s timeout followed each reward delivery to prevent continuous reward delivery. **Phase 1:** A single reward drop was placed at the spout at the start of each session to facilitate task engagement. Trials consisted of 85% CS+ (reward-predictive tone) and 15% CS− (non-predictive tone) presentations. Each CS was presented for 1.5 s, followed by a 2 s response window. On CS+ trials, reward was delivered upon the first lick within the response window. Licking during CS− trials had no programmed consequence. Mice advanced to the next phase upon responding to >40% of CS+ trials. **Phase 2:** No pre-placed reward was provided. CS+ and CS− cues were presented in 55% and 45% of trials, respectively. CS duration remained 1.5 s, with a 2 s response window. Mice advanced to Phase 3 if CS+ response rate exceeded 50%. **Phase 3:** Prior to each session, mice were habituated for 5 min to familiar environment. Training consisted of 55% CS+ and 45% CS− trials (1.5 s CS duration; 1 s response window). Mice progressed to testing only if they met the following criteria: (1) CS+ response rate exceeded CS− response rate by >30% for three consecutive days prior to testing; (2) CS+ responses remained stable. Stability was defined as an average change in CS+ response rate of less than ±5% across the final three training sessions.

### Social buffering of stress testing

Each mouse underwent 2–4 daily test sessions in a counterbalanced, pseudorandomized order across days. Sessions consisted of a baseline session and one or more of the following test conditions: control, control + social interaction (SI), restraint, restraint + SI. For experiments involving a familiar partner, mice completed 2 test sessions each. All other experiments were conducted using 4 testing sessions per mouse. Restraint sessions were never conducted on consecutive days to prevent cumulative stress effects. Depending on the experimental group, social interaction was conducted with either same-sex littermate cagemate (familiar partner), a novel sex- and age-matched conspecific (novel partner), or a familiar inanimate object. Both subject mice and their partners were separately habituated to the testing environment for 3–5 days prior to testing.

The test conditions were as follows: **Baseline**: Mice were placed in the familiar environment for 40 min. **Control**: Identical to Baseline. **Control + Social Interaction (SI)**: Mice spent 30 min alone in the familiar cage followed by 10 min of social interaction prior to testing. **Restraint**: Mice were restrained in ventilated plastic bags placed inside 50 mL Falcon tubes with a breathing hole for 30 min. A novel odor (essential oil: lemon, eucalyptus, orange, anise, almond or cinnamon) was applied to a cotton tip inside the tube. Odors were rotated across days to avoid habituation. After restraint, mice were placed alone in the familiar chamber for 10 min prior to testing. **Restraint + SI**: Mice underwent the same restraint procedure as above, followed by 10 min of social interaction with a partner before testing.

Each condition was immediately followed by a 45 min cued reward test session (~100 trials). Trials consisted of 65% CS+ and 35% CS− presentations. Inter-trial intervals (ITI) were pseudo-randomly varied between 17–33 s (mean ITI: 25 s). Each auditory cue was presented for 1.5 s and followed by a 1 s reward collection window, where a reward was delivered upon first lick.

### Social interaction scoring

Behavioral interactions were assessed for both the subject mouse (stressed or unstressed) and its unstressed partner. As described above, subject mice underwent 30 min of restraint stress and were then immediately placed in a familiar environment chamber with their designated partner for 10 min of free social interaction. All sessions were video recorded, and behavior was scored offline by trained experimenters. 10 different social and non-social behaviors were manually annotated: anogenital exploration (direct snout contact directed toward the partner’s anogenital region), body exploration (snout contact with other regions of the partner’s body), following (sustained locomotion behind the partner), allogrooming (grooming directed toward the partner), self-grooming, rearing (vertical exploration), supported rearing (vertical exploration with front paws in contact with wall) surveying (exploratory sniffing of the environment while walking or sitting), walking and sitting. Notably, no fighting behaviors were observed in any pairings, including both familiar and novel partners.

### Predictive modeling of behavioral features

We trained supervised machine learning models to predict stress-induced changes in response latency to reward predictive cues from behaviors occurring during 10-minute social interaction. Behavioral features were extracted from annotated video recordings and included the total duration, mean duration, frequency, mean inter-behavior interval, and frequency in the first and second half of the session. Each feature was attributed either to the stressed subject or to the unstressed partner. The target variable, latency change, was defined as the percent change in reward response latency relative to each animal’s individual baseline performance. All features were used in raw form (i.e., not normalized or standardized) to preserve their absolute behavioral magnitudes. Based on empirical testing, we found that three categories of features: total duration, mean duration, and frequency consistently contributed most to prediction accuracy. Features that did not improve model performance were excluded from further analysis.

To evaluate predictive accuracy, we used a feedforward neural network model (three hidden layers, 10 units each), selected as the best-performing model using MATLAB’s Regression Learner App. Model accuracy was evaluated using a leave-one-mouse-out (LOMO) cross-validation scheme, in which each animal was iteratively held out for testing while the model was trained on the remaining data. Predictive accuracy was quantified using Pearson’s correlation coefficient (*r*), coefficient of determination (*R2*), root mean squared error (RMSE), and mean absolute error (MAE). As a control, each behavioral feature was independently shuffled across animals, preserving their marginal distribution while disrupting any true association with the target. We then implemented a Random Forest regression model (400 trees; fitrensemble, MATLAB R2025a) to assess feature importance in predicting latency change. Relative feature importance scores were calculated using out-of-bag permutation-based impurity reduction. To evaluate the statistical significance of feature importance, we performed a permutation test (1,000 iterations) by randomly shuffling the latency change variable and retraining the model. Empirical *p*-values were computed as the proportion of null models in which the permuted importance exceeded the observed importance. Features with *p* < 0.05 were considered statistically predictive. All analyses were conducted using custom scripts and built-in functions in MATLAB R2025a.

### Receiver operating characteristic (ROC) analysis

Receiver-operating characteristic analysis was performed in MATLAB as described previously^60^, to assess the ability of latency to retrieve rewards and anticipatory licks to discriminate between baseline and stress conditions across familiar partner, familiar object and novel partner experiments. ROC curves and area under the curve (AUC) were computed using built-in MATLAB functions. AUCs were compared using a nonparametric bootstrap (10,000 resamples, with replacement).

### Surgery for fiber photometry recordings and optogenetic manipulations.

Mice were anesthetized with 5% isoflurane and placed into the stereotaxic apparatus (David Kopf Instruments) with mouse adaptor and lateral ear bars. Isoflurane was maintained at 1.5–2% during the entire surgery. Prior to surgery, analgesia was induced with subcutaneous administration of carprofen (5 mg/kg) and dexamethasone (2 mg/kg) in the back, along with local injection of lidocaine (0.1 ml, 0.5%, Lidor) at the level of the skull. A heating-pad was positioned underneath the animal to keep the body temperature at 37°C. Eye dehydration was prevented by topical application of ophthalmic gel. The skin above the skull was shaved with an electric razor and disinfected with 3 alternate swaps of 70% ethanol and iodine solution (10% Betadine) before an incision was made. Viral vectors were injected with a glass pipette attached to a microinjector (QSI, Stoelting, WI, USA). For fiber photometry recordings, DAT-Cre and VGAT-Cre mice were injected unilaterally into the left VTA with 400nl of pGP-AAV-syn-FLEX-jGCaMP8f-WPRE adeno-associated virus (Addgene, #162379-AAV5), at 2nl/sec, with the following coordinates (from Bregma): AP −3.4, ML −0.5, DV −4.4. The optical fiber (200 μm diameter) was placed 200 μm above the injection site and fixed with dental cement (Unifast Trad, GC Corporation, IL, USA). For optogenetic manipulations, DAT-Cre mice were injected with pAAV-EF1a-double floxed-hChR2(H134R)-EYFP-WPRE-HGHpA (Addgene, #20298-AAV5) and VGAT-Cre mice were injected with pAAV5-Ef1a-DIO eNpHR 3.0-EYFP (Addgene, #26966-AAV5) bilaterally into the VTA. Two optical fibers (200 μm diameter) were placed 200 μm above the injection site and fixed with dental cement.

Following any virus delivery, the pipette was left in place for at least 5 min before being slowly withdrawn from the brain. Following surgery, all mice received subcutaneous injection of anti-inflammatory drug carpofen (5mg/kg) for 3 days post surgery. Animals continued to be group housed and body weight was monitored daily for 3 days post-op to assess recovery. Behavioral experiments were carried out 4–6 weeks after surgery.

### Fiber photometry recordings and data analysis

Freely moving mice expressing Cre-dependent GCaMP8f were imaged 4–5 weeks post-surgery using a Neurophotometrics (San Diego, CA, USA) FP3002 fiber photometry system. Both fiber photometry and behavioral video recordings were acquired at 30 Hz. Fluorescence signals were evoked using alternating 470 nm (blue) and 415 nm (isosbestic UV) LED excitation. The light intensities at the tip of the patch cord were 50–70 μW for the 470 nm channel and 40–50 μW for the 415 nm channel. The isosbestic control signal was linearly fitted to the calcium-dependent signal via least-squares regression. ΔF/F was calculated as (F_signal – F_fit) / F_fit, where F_fit represents the fitted isosbestic signal. The ΔF/F traces were bandpass filtered between 0.02 and 10 Hz using a 4th-order Butterworth zero-phase filter to isolate biologically relevant transients.

Behavioral video recordings were synchronized with photometry data using Arduino-driven TTL pulses for the photometry system, paired with an LED light visible in the video, and custom MATLAB scripts. Behavioral epochs, including body sniffing, anogenital area sniffing, self-grooming, survey, walking, rearing, and sitting (as defined before), were manually annotated from synchronized videos. For each behavioral event, ΔF/F segments were extracted from −0.5 to +1 s relative to event onset. Baseline correction was performed by subtracting the mean ΔF/F signal from a pre-event baseline period (−0.5 to 0 s) for each trial, and data were aggregated across animals. Area under the curve (AUC) was computed for the post-onset window, with mean values and 95% confidence intervals calculated across events and animals. For data visualization mean ΔF/F traces were plotted with 95% confidence intervals, applying baseline correction. All intermediate and processed data were saved for subsequent statistical analysis.

### Optogenetic stimulation/inhibition

Optogenetic manipulations were performed in freely behaving mice using bilaterally implanted optical fibers targeting the VTA, as described above. Laser output was calibrated at the tip of the patch cord to 10 ± 1 mW for all experiments.

For DA neuron activation, a 473 nm laser was delivered in patterned bursts. Each stimulation epoch (1.5 s) consisted of three bursts of five pulses (5 ms pulse width, 20 Hz), separated by 250 ms intervals. Stimulation epochs were delivered every 15 s, resulting in a total of 36 stimulations over a 10 min session. This stimulation pattern was designed based on the temporal structure of affiliative interactions quantified in Fig. 1, in which mice exhibiting enhanced reward seeking engaged in ~30 social sniffing events of ~1.3 s duration, and was informed by previous work^61^.

For GABA neuron inhibition, a 532 nm laser was delivered as continuous illumination for 10 min.

### Closed-loop optogenetic inhibition based on real-time social proximity

Real-time, closed-loop optogenetic inhibition was implemented based on continuous tracking of freely moving mice. Body part positions were estimated using a multi-animal pose estimation model trained in SLEAP^62^ and integrated into a Bonsai^63^ workflow for real-time processing. Video was acquired using USB camera (1024 × 768 pixels, 30 Hz) and preprocessed to match the training conditions of the pose estimation model (grayscale conversion and spatial resizing). The trained model was used to extract the positions of multiple body parts for each animal in real time. Subsequent analysis focused on the Euclidean distance between the nose positions of the two animals, computed continuously for each frame. A proximity event was defined as a nose-to-nose distance below a fixed threshold (10 mm, empirically determined). Threshold crossings were converted into binary events and passed through a state-change filter to ensure that only the onset of each interaction triggered downstream signaling. Upon detection of a proximity event, a transistor–transistor logic (TTL) pulse was delivered via an Arduino microcontroller to a pulse generator (Master-8, A.M.P.I, Israel), which triggered laser for a fixed duration of 1.5 s, a time that was set based on average times of body sniffing interactions from our previous behavioral experiments. During this light delivery window, additional threshold crossings were ignored to prevent re-triggering. This pipeline enabled fully automated, real-time coupling of social interaction dynamics to optogenetic manipulation in freely moving pairs of mice. Laser output was calibrated at the tip of the patch cord to 10 ± 1 mW, using a 532 nm laser for photoinhibition.

For these experiments, both male and female VGAT-Cre mice expressing Cre-dependent halorhodopsin were bilaterally implanted above the ventral tegmental area (VTA) as described in the Surgery section. Mice were assigned to either closed-loop or yoked conditions. In the first batch of closed-loop mice (n=8), the total number of light delivery epochs was quantified for each animal (mean=61.4 events). A representative pattern (61 events) was selected to generate a yoked protocol. Light delivery epochs were reconstructed using a custom script in MATLAB and delivered via an Arduino-controlled trigger in the absence of a social partner. The same temporal pattern was applied to all animals in the yoked group. Following a 1-month interval, mice were crossed over between conditions such that each animal was tested under both closed-loop and yoked conditions.

### Perfusion

Mice were deeply anesthetized via intraperitoneal injection of ketamine (100 mg/kg) and xylazine (7 mg/kg) and transcardially perfused with 0.1 M phosphate-buffered saline (PBS, pH 7.4), followed by fixation with 4% formaldehyde (Sigma, HT501128–4L). Brains were post-fixed in the same fixative overnight at 4 °C, then transferred to 30% (wt/vol) sucrose (Sigma, S0389) for cryoprotection. Brains were embedded in O.C.T. compound (Tissue-Tek, CA, USA) and sectioned coronally (40 μm) using a cryostat (Leica Biosystems, CM1950S).

### Histology in the VTA

Sections were washed in PBS, mounted on slides, and coverslipped. Images were acquired using a fluorescence microscope (Leica Microsystems).

### Data collection

No statistical methods were used to pre-determine sample sizes; however, group sizes are consistent with similar published work. All mice were assigned pseudo-randomly to testing sessions and experimental groups. Although experimenters were aware of the experimental conditions, behavioral data in the cued-reward task as well as fiber photometry signals were collected automatically, minimizing potential observer bias.

### Statistical analysis

All experiments employed a within-subjects (repeated-measures) design, with each mouse tested across 2–4 pseudorandomized experimental conditions. Data from each condition were normalized to the individual mouse’s baseline performance for main and Extended Data figures, unless otherwise indicated, where raw data are shown. Data are presented as individual data points with means ± 95% confidence intervals or as individual data points or as proportions (pie charts), as appropriate. Each experiment was replicated in at least two independent batches of mice. Data were analyzed using GraphPad Prism 8.0.1 or MATLAB R2021a-2025a. Normality of the data was assessed using the Shapiro-Wilk test. Depending on the outcome, appropriate parametric tests (paired or unpaired Student’s t-test, one-sample t-test, ordinary one-way ANOVA with Bonferroni post hoc, ordinary two-way ANOVA, mixed-effects model [REML], or three-way ANOVA) or non-parametric tests (Mann-Whitney test, Wilcoxon matched-pairs signed-rank test, Friedman test, or Kruskal-Wallis test) were applied. Linear regression analyses were performed where indicated. The significance threshold was held at α = 0.05; all tests were two-sided; ns, not significant (p>0.05); *p<0.05, **p<0.01, ***p<0.001. Detailed statistical information, including exact group sizes, test statistics with degrees of freedom, and exact P values, is provided in Supplementary Table 1 (main figures) and Supplementary Table 2 (Extended Data figures).

## Supplementary Files

This is a list of supplementary files associated with this preprint. Click to download.

- SupplTable2ExtendedFigs13.pdf
- Supplvideo1FPbodysniffDARSIspeed0.8.mp4
- SupplTable1Figs13.pdf
- Supplvideo2FPbodysniffGABARSIspeed0.8.mp4
- Supplvideo3closedloopoptoexample.mp4

## Extended Data

## Data Availability

Additional data relating to the paper is available from the corresponding author on reasonable request.

## Code availability

Code relating to the paper is available from the corresponding author on reasonable request.
